# Human papillomavirus genotypes and factors associated with major cervical smear abnormalities in a sickle cell endemic area of Kisangani, Democratic Republic of the Congo

**DOI:** 10.1371/journal.pone.0350982

**Published:** 2026-06-10

**Authors:** Yvette Neema Ufoy Mungu, Joëlle Desreux, Elettra Bianchi, Burubu Lisi-Ankiene Junior, Roland Marini Djang’eing’a, Durkin Keith, Artesi Maria, Katenga Bosunga, Vincent Bours

**Affiliations:** 1 Department of Gynecology and Obstetrics, Faculty of Medicine and Pharmacy, University of Kisangani, Kisangani, Democratic Republic of the Congo; 2 Department of Biomedical Sciences, Faculty of Medicine, University of Liege, Liege, Belgium; 3 Department of Gynecology, Faculty of Medicine, University of Liege, Liege, Belgium; 4 Department of Anatomopathology, University Hospital of Liege, Liege, Belgium; 5 Department of Gynecology and Obstetrics, Faculty of Medicine, University of Kikwit, Kikwit, Democratic Republic of the Congo; 6 Laboratory of Analytical Pharmaceutical Chemistry, CIRM, Faculty of Medicine, University of Liège, Liege, Belgium; 7 Department of Pharmacy, Faculty of Medicine and Pharmacy, University of Kisangani, Kisangani, Democratic Republic of the Congo; 8 Department of Biomedical and Preclinical Sciences, Human Genetics, GIGA Research, Faculty of Medicine, University of Liege, Liege, Belgium; University of Bunia; Centre Interdisciplinaire de Recherche Translationnelle en Medecine et Sciences de la Sante (CIRTMSS), CONGO, THE DEMOCRATIC REPUBLIC OF THE

## Abstract

**Background:**

Cervical cancer due to high-risk human papillomavirus (HR-HPV) is the leading gynecological cancer in Sub-Saharan Africa and in the Democratic Republic of the Congo (DRC), where approximately one-quarter of people carries the sickle cell allele. This study aims to identify the HR-HPV genotypes and factors associated with major cervical abnormalities among women living in the sickle cell endemic area of Kisangani, DRC.

**Methods:**

This hospital-based cross-sectional study consisted to a face-to-face and semi-structured questionnaire for collecting data of the socio-demographic, clinical and sexual behaviors characteristics of participants. The sickle cell status was confirmed using liquid chromatography coupled with mass spectrometry. Cervical smears were collected using the ThinPrep^®^ Pap-test solution (Hologic Inc, Marlborough, USA). The HR-HPV molecular analysis was performed using the Cobas^®^ 6800 system (Roche Molecular Systems, Somerville, USA) for real time polymerase chain reaction (RT-PCR) and the Oxford Nanopore platform (Oxford Nanopore Technologies, Oxford, England) for the viral genome sequencing. Cytological analyses were performed using the ThinPrep 5000 processor (Hologic), and the results were reported according to the 2014 Bethesda System classification. The logistic regression model was used to estimate the adjusted Odd ratio (aOR) during the risk factor assessments.

**Results:**

Among 712 women enrolled, the prevalence of HR-HPV was 28.4% (95% CI: 25.1–31.7). HPV35, HPV52 and HPV31 were the most common genotypes; while the sequencing identified two new variants of HPV103 and HPV223. The following factors were associated with HR-HPV infection: (i) age between 25 and 34 years (aOR: 2.10, 95% CI:1.22–3.69); (ii) being unmarried (aOR: 1.54, 95% CI: 1.05–2.27) and (iii) HIV-positive status (aOR: 14.85, 95% CI: 2.36–288.25). No correlation was observed between hemoglobin AS (HbAS) status and HR-HPV infection or cytological abnormalities. However, women aged 45–54 years (aOR: 3.8, 95% CI: 1.34–11.65), those using intravaginal herbal (aOR: 3.59, 95% CI: 1.26–11.28) and those infected with HPV33 (aOR: 8.45, 95% CI: 1.68–34.05) were more likely to present major cervical abnormalities in our series.

**Conclusion:**

Our study shows a high prevalence of cervical HR-HPV infection in Kisangani, DRC. HPV35, HPV52, and HPV31 are the three most common genotypes, while novel variants of HPV types 103 and 223 were identified. Our findings also highlight that advanced age, HPV33 infection and intravaginal herbal use are the main factors associated with major cervical abnormalities. Although HbAS has not been associated with HR-HPV infection or cervical lesions, further studies are needed to determine this association in women with sickle cell disease (HbSS).

## Introduction

Human papillomavirus (HPV) is one of the most common sexually transmitted infections worldwide, affecting more than 80% of men and women at some point in their lifetimes [1,2]. Since 1985, many studies have firmly proven this virus to be the most important etiological agent for the development of cervical intraepithelial neoplasia (CIN) and invasive cervical cancer [1,3].

Cervical cancer is the fourth deadliest cancer in women worldwide, with an estimated 660,000 new cases and 350,000 deaths in 2022, and it is the leading cause of female cancer in the Democratic Republic of the Congo (DRC) [4]. In 2023, the country recorded 9,000 new cases, resulting in 6,000 deaths [5,6].

The prevalence and distribution of HPV varies across regions and countries. Among women with normal cytology, the global prevalence of cervical infection was estimated at approximately 11.7% and 9.9% respectively in 2010 and 2019 [7,8]. The highest HPV prevalence in these women was observed in Sub-Saharan Africa (SSA) (24% in 2010, estimated to 31.9% in 2023) and in Oceania (22% in 2023) [7,9]. European women are more likely to be infected with HPV16 and HPV18 than women in SSA [10] and Asia [11–13].

High-risk (HR) HPV infection causes low-grade cervical lesions, some of which progress to high-grade (precancerous) lesions, which rarely regress [14,15]. Hence, in 2020, the World Health Organization (WHO) initiated a global strategy to eliminate cervical cancer as a public health problem by 2030. This includes vaccination against HPV, screening, and effective treatment of precancerous lesions and cervical cancer [16].

Personalized screening strategies based on individual susceptibility are encouraged to enable the implementation of targeted preventive measures [17]. Accordingly, the screening guidelines for individuals with congenital or acquired immunodeficiency have been updated [18].

Sickle cell disease (SCD) is an autosomal recessive disease endemic to SSA, the Mediterranean Basin, the Middle East, and India that is characterized by the presence of hemoglobin S. Homozygotes (HbSS) are symptomatic and show an increased susceptibility to infections. In contrast, heterozygotes or people with hemoglobin AS (HbAS) are asymptomatic and have a life expectancy similar to that of controls [19,20].

Between 25% and 30% of the population in the DRC carries the sickle cell allele and, in Kisangani, 24% of women are HbAS [20,21]. These individuals have a natural resistance to severe forms of malaria due to their immune response [19,20]. Other studies have found a high susceptibility to urinary tract infections among HbAS people [22,23]. However, their immune response has not been sufficiently studied in relation to other pathogens present in areas affected by SCD, notably HPV.

Genotypes of HR-HPV circulating in the DRC are poorly documented [6,24]. However, Mutombo et al. [25] and Muwonga et al. [26] reported an HR-HPV prevalence ranging from 24.8% to 36.9%, with a predominance of HPV68 and HPV52. Previous studies have been conducted either on the general population [25,27] or on women living with Human Immunodeficiency Virus (HIV) [26,28]. However, most did not address the correlation between HPV type and corresponding cervical abnormalities [25–27].

In 2015, the DRC has developed a strategy to eliminate cervical cancer as a public health problem [29]. However, almost 10 years later, the HPV vaccine has still not been implemented, and screening often remains opportunistic [29,30]. Knowledge about the HPV genotypes circulating in Kisangani and whether HbAS women are susceptible to developing cervical abnormalities could enrich the existing data and enable the adaptation of cervical cancer prevention strategies in the DRC. This study aims to: *(i)* identify the HR-HPV genotypes present in cervical smears from women living in the sickle cell endemic area of Kisangani, DRC, (*ii*) determine the risk factors for HR-HPV infection, and (*iii*) identify factors associated with major cervical abnormalities in our cohort.

## Materials and methods

### Study design and settings

This hospital-based cross-sectional study was carried out among volunteers consisting to face-to-face and semi-structured questionnaire, blood draws for laboratory tests designed to identify women with sickle cell trait, and Pap smear testing for the detection of HR-HPV infection and cervical abnormalities. Data were collected prospectively in six healthcare facilities (the Kisangani University Clinics, the Kabondo General Referral Hospital, the Marie Reine Referral Health Center, the Millennium Polyclinic of Kisangani, the Delvaux Bolila Hospital Center, and the Reverend Mokili Referral Health Center) in the city of Kisangani, the capital of Tshopo Province in northeastern DRC, from February 17, 2023 to February 18, 2024. Those facilities were chosen based on their geographical accessibility, high attendance rates, and proximity to neighboring municipalities, with the aim of diversifying the origin of the respondents as much as possible. The STROBE guideline was used for reporting this study.

### Population sampling and recruitment

We organized awareness campaigns that included free gynecological consultations with screening for cervical cancer and SCD. In the hospital waiting room, a physician first provided the women general information about the study and then sought their free and informed consent. A nurse distributed information sheets and informed consent forms written in French and in the local languages (Lingala and Swahili) to the women.

The respondents received detailed information and counseling regarding sickle cell and HIV screening in the doctor’s office. After providing a duly signed written consent form, the socio-demographic characteristics and sexual behaviors of the participants were recorded on a data collection form. The doctor proceeded then with the gynecological examination. The cervical specimen for liquid-based cytology was collected using a combined cytobrush and endocervical brushes (Rovers Medical Devices, Netherlands, Cervex Brush^®^) after inserting a single-use bivalve speculum within a woman’s vagina while she lay in the lithotomy position on an examination bed. The sample was immersed and rinsed in a vial containing 20 ml of ThinPrep^®^ Pap-test solution (PreservCyt^®^). After the clinical examination, the respondent was referred to the laboratory for SCD screening using the HemoType SC^TM^ rapid test. During the test, a second sample of two to three drops of blood was collected on blotting paper, assigned an identification number identical to that of the vial, and dried. HIV testing was performed immediately following SCD screening.

We stored the cervical smears in a refrigerator at temperatures between 2 and 8° Celsius, in accordance with the manufacturer’s recommendations [31]. The samples were stored temporarily at the Kisangani University Clinics while awaiting shipment to the National Institute for Biomedical Research (the INRB in French) in Kinshasa. There, the smears were stored in a refrigerator for 7–10 days, pending shipment to Belgium. In the meantime, the blood samples dried on blotting paper were shipped by DHL, in separate envelopes. The results of the analyses were available within 4–6 weeks after their arrival in Belgium.

### Eligibility criteria

Eligible participants were women aged 25 years and older who had voluntarily agreed to participate in the study, had reported having sexual intercourse, and had no history of total hysterectomy or local treatment for precancerous or cancerous cervical lesions. Exclusion criteria included pregnancy, postpartum status, current or recent menstruation, current vaginal treatment, sexual intercourse within 48 hours prior to the sampling, and acute cervicitis.

### Sample analysis and measures

Cervical smears and dried blood samples for confirmation of SCD were analyzed at the laboratory of the University Hospital Center of Liege, Belgium. This laboratory has been accredited for genetic, anatomical pathology, and cytopathology analysis since 2005 [32]. It should be noted that HIV serological screening, using the current testing algorithm in force in the DRC, was performed immediately in the field on whole blood collected via finger prick [33].

### Cytological analyses

The ThinPrep vial containing the sample was placed in the ThinPrep 5000 processor with Autoloader system (Hologic Inc, Marlborough, USA) [34] for analysis. The slides were examined by a team of qualified cytotechnicians, and the results were validated by cytopathology department supervisors. The cytology results were reported according to the 2014 Bethesda system nomenclature [35]. Squamous cell abnormalities were categorized as follows: negative for intraepithelial lesion or malignancy (NILM), atypical squamous cells of undetermined significance (ASC-US), low-grade squamous intraepithelial lesion (LSIL), high-grade squamous intraepithelial lesion (HSIL), and atypical squamous cells that cannot exclude HSIL (ASC-H). Glandular cell abnormalities were categorized as follows: atypical glandular endocervical cells not otherwise specified (AGC-NOS) and atypical glandular endocervical cells, favor neoplastic (AGC-ecc favor neoplastic).

### HPV test and genotyping

The HPV test was performed using the Cobas^®^ 6800 system (Roche Molecular Systems, Somerville, USA). The test uses amplification of target desoxyribonucleic acid (DNA) by polymerase chain reaction (PCR) and nucleic acid hybridization for the detection of 14 HR-HPV types [36]. Aliquots of 1–2 ml were taken from each initial ThinPrep vial and transferred to deep-well tubes. The Roche 6800 pre-analytical instrument was used to process the samples before they were analyzed on the Cobas 6800 system. The 14 detected HR-HPV are divided into 12 types of carcinogenic HPV in group 1, namely HPV 16, 18, 31, 33, 35, 39, 45, 51, 52, 56, 58, and 59, as well as HPV 68 and 66, classified, respectively, in groups 2A and 2B of carcinogenic HPV types [37]. The final categorical results were recorded as follows: HR-HPV negative, HPV16 positive, HPV18 positive, positive for other HR-HPV (the remaining 12 types), and invalid (sample not evaluable) [36].

A detailed description of the PCR and rolling circle-based nanopore sequencing, as well as a more detailed examination of the resultant sequences, can be found in Artesi et al. [38]. Briefly, HR-HPV were PCR amplified using two amplicon pools designed with primalscheme (https://github.com/aresti/primalscheme); these targeted all HR-HPV (16, 18, 31, 33, 35, 39, 45, 51, 52, 56, 58, 59, 66, and 68). The resultant PCR products (~1.5 kb in length) were barcoded with the Oxford Nanopore Native Barcoding Kit 96 V14 kit (Oxford Nanopore Technologies, Oxford, England), following the manufacturer’s instructions, and sequenced with R10.4.1 flow cells. Additionally, a subset of the samples underwent rolling circle amplification (RCA) using the Qiagen REPLI-g kit. Amplified DNA was debranched with T7 endonuclease I and barcoded with the Oxford Nanopore Native Barcoding Kit 96 V14 kit. Both types of libraries were sequenced on R10.4.1 MinION or PromethION flow cells (Oxford Nanopore Technologies, Oxford, England). In the case of the RCA libraries, nanopore adaptive sampling was used to enrich the viral reads by selecting against those that mapped to the human genome. For both approaches, base calling was performed with Dorado (https://github.com/nanoporetech/dorado) using the Super Accurate model: dna_r10.4.1_e8.2_400bps_sup@v5.0.0. Demultiplexing required barcodes on both ends of the molecule.

Consensus sequences were generated using medaka (https://github.com/nanoporetech/medaka), requiring at least 15X coverage across > 50% of the HPV genome, and where coverage fell below 15X, the region was masked with Ns (unknown DNA bases).

### Sickle cell disease diagnostic

The rapid diagnosis of SCD was performed using HemoTypeSC (Silver Lake Research Corporation, 1300 West Optical Drive, Azusa, CA 91702, USA). It is a competitive lateral flow immunoassay that uses monoclonal antibodies to detect hemoglobin A, S, and C in a 1.5 µL whole blood sample at the point of care [39]. In Belgium, the clinical hematology laboratory used the triple quadrupole mass spectrometer system TQ5500 (Sciex, Nieuwerkerkerlaan, Netherlands) for liquid chromatography, coupled with mass spectrometry (LC-MS) analysis following the manufacturer’s technical specifications, as described for analyzing blood samples taken on blotting paper [40].

### Operational definition of variables

To facilitate the analysis, the 14 HPV types detected by Cobas [36] were all considered HR-HPV. An HPV sequence was considered a novel type if the L1 sequence showed > 10% divergence from its closest match in the PAVE Reference Genomes (https://pave.niaid.nih.gov/explore/reference_genomes/human_genomes) by the BLAST. We referred to situations in which HR-HPV coexisted with other HPV types as co-infection. In these cases, the other HPV genotypes were classified as probably high-risk HPV (pHR-HPV), considered possibly oncogenic, or low-risk HPV (LR-HPV), considered non-oncogenic, based on data from the International HPV Reference Center (https://www.hpvcenter.se/) and the McBride AA study [14]. The results of the cytological analyses were grouped into three categories [18]: normal cytology (NILM and inflammatory smears); abnormal cytology, including minor abnormalities (ASC-US and LSIL) and major abnormalities (ASC-H, HSIL, AGC-Nos, and AGC-in favor of neoplasia).

### Sample size

The sample size was calculated using PASS 2023, version 23.01 software. This calculation was based on the prevalence of HPV infection of 28.2% (95% CI: 26.1–30.3%) [25,27] and the proportion of HbAS people estimated at 25% in the DRC [20]. We considered a prevalence of 5% of HPV types 16 and 18 in Congolese women [25].

Since no prior studies on HPV infection in HbAS women were identified, the sample size was determined mid-study using data from the first 307 participants (248 HbAA and 59 HbAS). Based on the existing data, increasing the sample sizes to 175 and 522 for HbAS and HbAA women, respectively, was important to acquire a minimum of 697 participants. This adjustment was necessary to achieve 80% power to detect HR-HPV genotypes, with α = 0.05. Due to logistical and financial constraints, only 130 HbAS women were recruited instead of the planned 175. However, a minimum sample size of 522 women with HbAA was achieved. Accounting for a 10% rate of invalid smears, the minimum sample size required to differentiate HR-HPV types was calculated at 767 women. Finally, we examined 736 women who met the criteria for cervical sampling, including 130 HbAS, 582 HbAA, and 24 who did not consent to screening for SCD and/or HIV infection.

### Statistical analyses

Data were entered using Microsoft Excel 2019 and analyzed with R software (version 4.4.3). Categorical variables were summarized using absolute and relative frequencies, while continuous variables were described using the mean ± standard deviation (SD). Two HbSS women were excluded due to their insufficient numbers to facilitate statistical analysis. Comparisons of participant characteristics by sickle cell status (HbAS vs HbAA) were conducted using Fisher’s exact test for categorical variables and the Wilcoxon rank-sum test for continuous variables. Determinants of HR-HPV infection and cervical lesions were first assessed using univariate analyses. Variables with *p-value* ≤ 0.20 were considered eligible for inclusion in the multivariable logistic regression model. A forward stepwise selection procedure based on the Akaike Information Criterion (AIC) was then applied to identify the final model. In cases of multicollinearity, one of the correlated variables was excluded from the model. Associations between explanatory variables and outcomes were reported as adjusted odds ratios (aORs) with corresponding 95% confidence intervals (CIs). A two-sided *p-value* < 0.05 was considered statistically significant.

### Ethics statement

The study was conducted in accordance with the General Data Protection Regulation (GDPR) and the principles of the Declaration of Helsinki. It was initially approved by the Ethics Committee of the University of Kisangani, DRC (approval number: UNIKIS/CER/023/2022) and subsequently by the Ethics Committee of the School of Public Health at the University of Kinshasa (approval number: ESP/CE/65/2025) for HPV genotyping and a possible study of the integration of viral DNA into the host genome. All participants freely provided written signed consent to participate in the study and to have their samples transported abroad. The consent process included information about the storage and potential future use of de-identified genetic data (viral and host), in accordance with the GDPR guidelines. Authorization to transport the samples to Belgium was obtained from the National Institute for Biomedical Research. To guarantee the confidentiality of all respondent information, research codes were assigned to all collected data and the corresponding Excel spreadsheet was stored on a password-protected computer.

The principles of beneficence and nonmaleficence were applied. We provided genetic counseling to single women who tested HbAS and screened the children of HbAS mothers upon request. The laboratories received anonymous samples. Women who tested positive for HIV for the first time were referred to the local National HIV/AIDS Control Program branch for treatment. The participants were contacted again and treated eventually as soon as the laboratory results were available.

### Inclusivity in global research

Additional information regarding ethical, cultural, and scientific considerations specific to inclusivity in global research is included in the supplementary information (S2 Appendix).

## Results

### Selection of respondents

**Fig 1:**
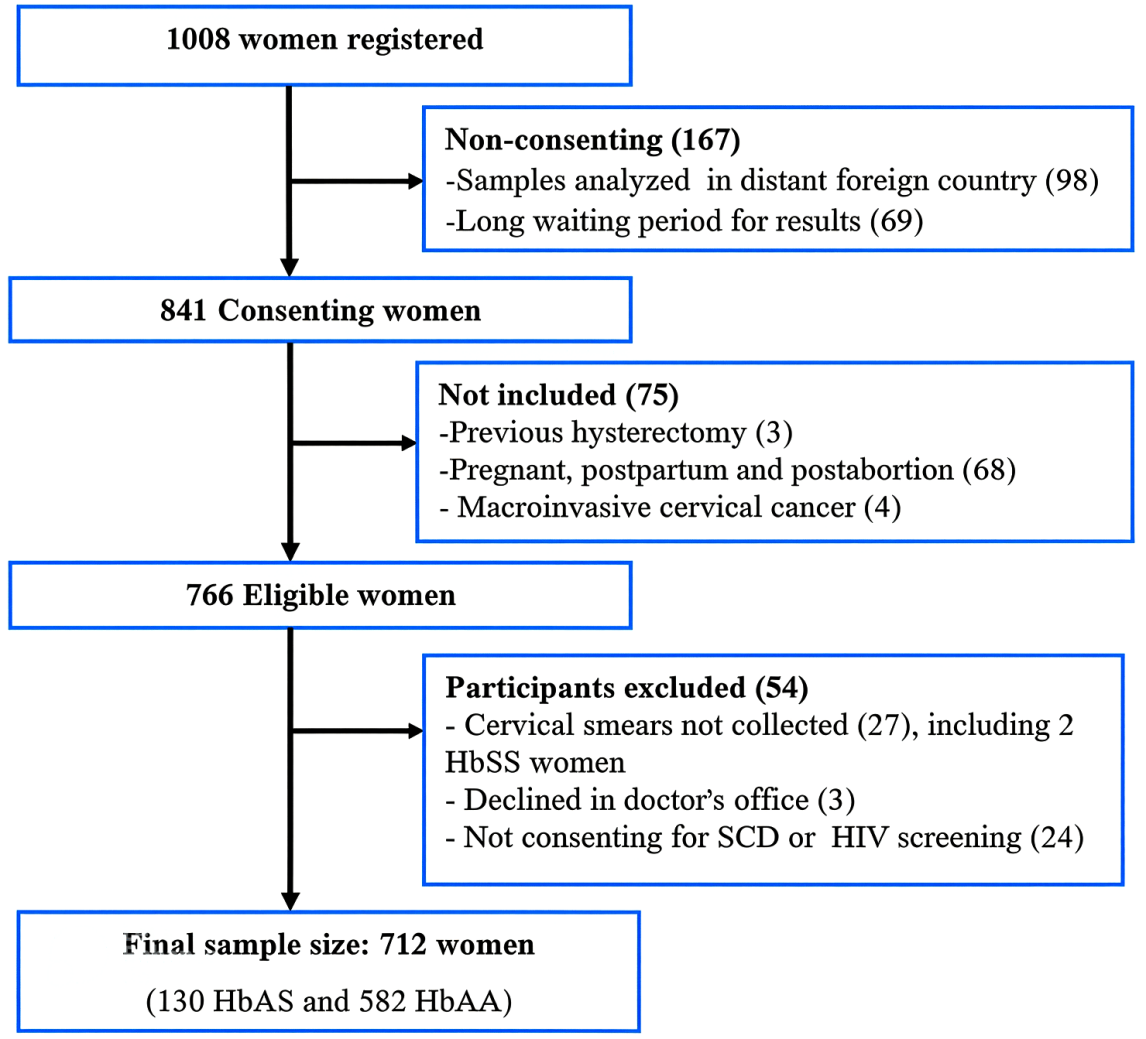
Selection of respondents.

Initially, 1,008 women participated in the free gynecological consultation campaign. After receiving general information, 167 women did not consent. Seventy-five consenting women out of 841 were excluded for various reasons. Among the 766 eligible women, 3 declined in the doctor’s office, and 24 did not consent to the blood tests. Twenty-seven eligible participants, including two HbSS women, did not show up for the rescheduled cervical sampling. They were not in optimal condition (menstruation, recent intercourse, or vaginal ovum) when they gave their consent. Therefore, our final sample included 712 participants (Fig 1).

### Socio-demographic characteristics of respondents

The participant’s socio-demographic characteristics are presented in Table 1. The mean age of the respondents was 38.6 ± 11.2 years.

**Table 1:** Socio-demographic characteristics of respondents.

Socio-demographic characteristics	Frequency (%) N = 712
**Age groups (years)**	
25–34	302 (42.4%)
35–44	195 (27.4%)
45–54	140 (19.7%)
55–64	62 (8.7%)
≥65	13 (1.8%)
Mean age ± SD	38.6 ± 11.2
**Education level**	
None	30 (4.2%)
Primary	147 (20.7%)
Secondary	366 (51.4%)
Superior and university	169 (23.7%)
**Profession**	
None	359 (50.4%)
Remunerated	74 (10.4%)
Nonremunerated	279 (39.2%)
**Marital status**	
Single	178 (25.0%)
Married	458 (64.3%)
Divorced / Widowed	76 (10.7%)
**Sampling site**	
The Kisangani University Clinics	220 (30.9%)
Reverend Mokili Referral Health Center	148 (20.8%)
Marie Reine Referral Health Center	43 (6.0%)
The Millennium Polyclinic of Kisangani	83 (11.7%)
The Delvaux Bolila Hospital Center	163 (22.9%)
Kabondo General Referral Hospital	55 (7.7%)
**Parity**	
0	109 (15.3%)
1–2	166 (23.3%)
3–5	253 (35.5%)
≥6	184 (25.9%)
Mean parity ± SD	3.7 ± 2.8

### Respondents’ sexual behaviors, cervical cytology and blood tests

The results recorded in Table 2 show that 63.2% of the participants had their first sexual intercourse before the age of 18, and 89.9% of the women had had at least two sexual partners in their lifetime. Additionally, 29.6% of the women used traditional herbs inside the vagina. Abnormal cytology was detected in 9.7% of the samples. The sample included 18.3% of HbAS and 1.3% HIV-positive women.

**Table 2:** Sexual behaviors, cervical cytology and blood tests.

Characteristics of respondents	Frequency (%) N = 712
**Age at first intercourse (years)**	
>17	262 (36.8%)
≤17	450 (63.2%)
Mean age at first intercourse ± SD	16.9 ± 2.9
**Number of lifetime sexual partners**	
1	72 (10.1%)
≥2	640 (89.9%)
**Intravaginal herbal use**	
Yes	211 (29.6%)
No	501 (70.4%)
**Previous cervical screening smear**	
Yes	29 (4.1%)
No	683 (95.9%)
**History of oral contraception**	
None	573 (80.5%)
<3 months	91 (12.8%)
≥3 months	48 (6.7%)
**Cytology results**	
NILM, Inflammatory smears	623 (87.5%)
ASC-US, LSIL	52 (7.3%)
AGC-NOS, AGC-ecc favor neoplastic, ASC-H, HSIL	17 (2.4%)
Invalid smears	20 (2.8%)
**Sickle cell status**	
HbAS	130 (18.3%)
HbAA	582 (81.7%)
**HIV serostatus**	
Positive	9 (1.3%)
Negative	703 (98.7%)

### Human papillomavirus test results

Partial genotyping using the Cobas 6800 detected HR-HPV in 200 participants (28.4%, 95% CI: 25.1–31.7), with a predominance of no-16/18 genotypes (24.4%, 95% CI: 21–27). HPV types 16 and 18 were detected in 3.5% (95% CI: 2.3–5.2), and 3.1% (95% CI: 2–4.7) of cases, respectively. The table 3 presents the distribution of HPV types identified in the 128 sequenced samples: 63.3% (95% CI: 54.3–71.5) of the samples tested positive for at least one HR-HPV type covered by Gardasil 9. The prevalence and the genotypes of HPV were not correlated with HbAS status (all *p-value* ≥ 0.05).

**Table 3:** Distribution of HPV genotypes in a sickle cell endemic area of Kisangani, DRC.

HPV genotypes	Total		Sickle cell status		
	N = 128	95% CI	HbAA N = 98	HbAS N = 30	*p-value*
**High-risk HPV**					
At least HPV type 16 or 18	22 (17.2%)	11.3–25.1%	14 (14.3%)	8 (26.7%)	0.12
At least HPV type 16,18, 31, 33, 45, 52 or 58	81 (63.3%)	54.3–71.5%	61 (62,2%)	20 (66.7%)	0.7
At least HPV type 35, 39, 51, 56, 59, 66 or 68	75 (58.6%)	49.5–67.1%	55 (56.1%)	20 (66.7%)	0,3
**Co-infections**					
HR-HPV/Other types (All)	39 (30.5%)	23–39%	30 (30.6%)	9 (30.0%)	0.9
HR-HPV/pHR-HPV	14 (10.9%)	6.3–18%	12 (12.2%)	2 (6.7%)	0.5
HR-HPV/LR-HPV	30 (23.4%)	17–32%	22 (22.4%)	8 (26.7%)	0.6
HR-HPV/ Uncategorized	8 (6.3%)	2.9–12%	6 (6.1%)	2 (6.7%)	0.9

**pHR-HPV:** HPV types 30, 34, 53, 67, 69, 70 and 82; **LR-HPV:** HPV types 40, 42, 44, 54, 61, 62, 72, 74, 81, 83, 86, 87, 89 and 90; **Uncategorized:** HPV types 5, 103, 103 Like-novel, 223, 223 Like-novel and 226

### High-risk HPV genotypes in women with or without sickle cell trait

**Fig 2:**
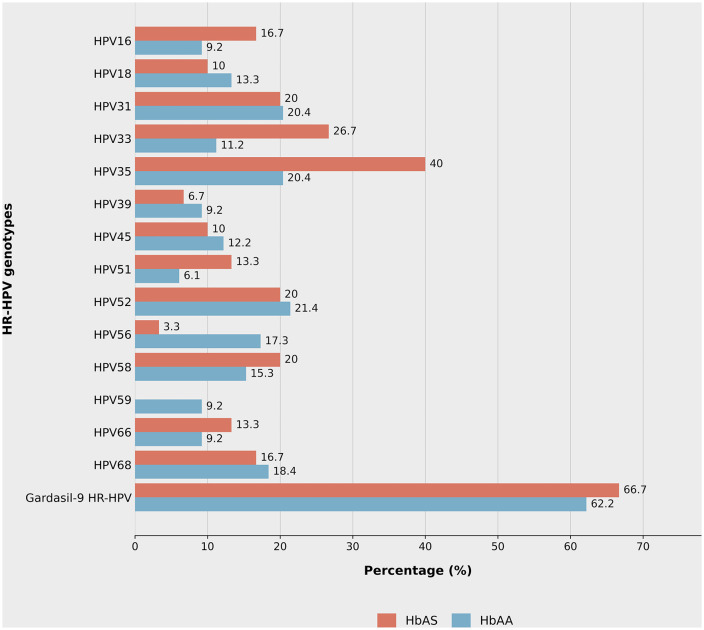
High-risk HPV genotypes in women with or without sickle cell trait in Kisangani, DRC.

The 14 types of HR-HPV were detected in the following order of frequency: 32 samples HPV35 (25%), 27 samples HPV52 (21.1%), 26 samples HPV31 (20.3%), 23 samples HPV68 (18.0%), 21 samples HPV58 (16.4%), 19 HPV33 samples (14.8%), 18 HPV56 samples (14.1%), 16 HPV18 samples (12.5%), 14 HPV16/HPV39 samples (10.9% for each genotype), 13 HPV66 samples (10.2%), 12 HPV45 samples (9.4%), 10 HPV51 samples (7.8%), and 9 HPV59 samples (7.0%). The 72 samples that tested positive for HR-HPV using the Cobas 6800 system but were not sequenced included 11 HPV16-positive samples, 6 HPV18-positive samples, and 55 samples containing no-16/18 genotypes. In descending order of frequency: HPV35, HPV33, and HPV31/HPV52/HPV58 predominated in HbAS women while HPV52 and HPV35/HPV31 were more frequent in controls (Fig 2).

### Potential new variants of HPVs 103 and 223 in cervical smears from women in Kisangani, DRC

Table 4 describes the profile of three women with HbAA, aged 30–51 and HIV-negative, in whom two variants of HPV that appeared to be new were identified. Indeed, our sequencing identified two novel HPVs (< 90% similarity to existing HPV L1): The first was most closely related to HPV103, with 84% identity, and was observed in two independent samples; the second matched HPV223, with 78% identity. The molecular clones were submitted to the International HPV Reference Center for validation in December 2025.

**Table 4:** Age and biological profile of carriers of potential new HPV in Kisangani, RDC.

Sample	Age (years)	New variants	Healthcare facility	Cytology	Associated HR-HPVs	Other associated HPVs
1	51	HPV103 like-novel	The Kisangani University Clinics	NILM	HPV31 and HPV35	HPV74
2	30	HPV103 like-novel	Marie Reine Referral Health Center	ASC-US	HPV51, HPV59, HPV66, and HPV68	HPV61
3	39	HPV223 like-novel	The Kabondo General Referral Hospital	NILM	HPV18, HPV31, HPV39, HPV52, HPV56, and HPV58	HPV61

NILM: negative for intraepithelial lesion or malignancy; ASC-US: atypical squamous cells of undetermined significance

### Factors associated with high-risk HPV infection

Determinants of HR-HPV infection were as follows: between 25 and 34 years old (aOR: 2.07, 95% CI:1.2–3.67; *p-value* = 0.01), to be unmarried (aOR: 1.51, 95% CI: 1.02–2.23; *p-value* = 0.039), and HIV-positive status (aOR: 14.85, 95% CI: 2.36–288.25; *p-value* = 0.015). In addition, there was an approximately 13 times greater risk of HR-HPV infection among women with abnormal cytology (aOR: 12.83, 95% CI: 7.04–24.88; *p-value* < 0.001) than in controls (Table 5).

**Table 5:** Factors associated with high-risk HPV infection in opportunistic screening among women in Kisangani, DRC.

Variable	Univariable analysis			Multivariable analysis		
	OR	95% CI	*p-value*	aOR	95% CI	*p-value*
**Age groups (years)**						
45–54 (Ref)	—	—	—	—	—	—
25–34	1.75	1.11–2.82	0.019	2.07	1.2–3.67	**0.01**
35–44	1.20	0.72–2.01	0.494	1.51	0.84–2.75	0.174
55–64	1.36	0.67–2.68	0.382	1.20	0.54–2.6	0.652
≥65	1.56	0.4–5.16	0.482	1.02	0.22–3.9	0.98
**Education level**						
Superior and University(Ref)	—	—	—	—	—	—
None/primary	1.22	0.77–1.96	0.4	1.74	0.96–3.17	0.07
Secondary	1.02	0.68–1.55	0.916	1.22	0.75–2	0.423
**Marital status**:unmarried	1.37	0.97–1.91	0.071	1.51	1.02–2.23	**0.039**
**Use of intravaginal plants**	1.36	0.96–1.93	0.086	1.18	0.78–1.76	0.427
**HIV-positive women**	9.12	2.18–61.58	0.006	14.85	2.36–288.25	**0.015**
**HbAS women**	1.43	0.95–2.14	0.081	1.38	0.87–2.15	0.168
**Abnormal cytology**	13.11	7.28–25.17	<0.001	12.83	7.04–24.88	**<0.001**

aOR: Adjusted odds ratio; CI: confidence interval; Abnormal cytology (≥ ASC-US); Ref: Reference group

### Factors associated with abnormal cervical cytology

We found that 69 women (9.7% of cases) had abnormal cytology (≥ ASC-US). An HR-HPV infection was the only factor associated with abnormal cytology (aOR: 12.74, 95% CI: 7.02–24.57; *p-value* *<* 0.001) as shown in Table 6. Details on cytology results for each HPV type can be found in S4 Appendix.

**Table 6:** Factors associated with abnormal cytology in opportunistic screening among women in Kisangani, DRC.

Variable	Univariable analysis			Multivariable analysis		
	OR	95% CI	*p-value*	aOR	95% CI	*p-value*
**Age at first intercourse**						
≤ 17 years (Ref)	–	–	–	–	–	–
>17 years	0.62	0.35–1.06	0.089	0.68	0.36–1.22	0.203
**Use of intravaginal plants**						
No (Ref)	–	–	–	–	–	–
Yes	1.83	1.09–3.03	0.02	1.54	0.87–2.7	0.132
**Sickle cell status**						
HbAA (Ref)	–	–	–	–	–	–
HbAS	1.49	0.81–2.63	0,18	1.23	0.63–2.29	0.536
**HIV serostatus**						
Negative (Ref)	–	–	–	–	–	–
Positive	3.07	0.44–13.63	0.175	0.82	0.11–3.93	0.816
**HR-HPV infection**						
HR-HPV negative (Ref)	–	–	–	–	–	–
HR-HPV positive	13.11	7.28–25.17	<0.001	12.74	7.02–24.57	**<0.001**

aOR: adjusted odds ratio; CI: confidence interval; Ref.: Reference group

### Factors associated with major cervical abnormalities

Seventeen women (2.4%) had major cytological abnormalities (ASC-H, HSIL, AGC-Nos, or AGC-ecc favor neoplastic). Our study (Table 7) revealed that the risk of major cervical abnormalities was significantly higher in patients aged 45–54 (aOR: 3.8, 95% CI: 1.34–11.65; *p-value =* 0.014), among those using intravaginal herbal products (aOR: 3.59, 95% CI:1.26–11.28; *p-value =* 0.020), or if infected with HPV33 (aOR: 8.45, 95% CI: 1.68–34.05; *p-value =* 0.004).

**Table 7:** Factors associated with major cervical abnormalities in women in Kisangani, DRC.

Variable	Univariable analysis			Multivariable analysis		
	OR	95% CI	*p-value*	aOR	95% CI	*p-value*
Age 45–54 (years old)	3.41	1.29–9.52	0.014	3.80	1.34–11.65	**0.014**
Intravaginal traditional plants	4.54	1.7–13.33	0.003	3.59	1.26–11.28	**0.020**
HPV33	9.00	1.94–31.11	0.001	8.45	1.68–34.05	**0.004**
HPV35	4.87	1.08–15.96	0.017	3.82	0.75–14.92	0.071
HPV51	4.72	0.25–27.41	0,153	2.86	0.13–21.38	0.379
HPV52	3.54	0.54–13.47	0.105	1.65	0.21–8.48	0.576
HPV58	4.69	0.71–18.28	0.050	1.94	0.24–9.84	0.467
HPV18	5.60	0.84–22.19	0.030	3.73	0.49–17.92	0.135

aOR: adjusted odds ratio; CI: confidence interval

## Discussion

This observational study assessed the potential role of the sickle cell trait (HbAS) as a factor associated with infection by HR-HPV, as well as with cervical cytological abnormalities, while accounting for sociodemographic characteristics and sexual behaviors. Women from the general population were recruited in a hospital setting, and their HIV serostatus was documented.

### Key results

The proportion of HbAS women in our sample (18.3%) was slightly lower than that reported in Kisangani (24%) and below the estimated prevalence of 25–30% in the DRC [20,21], probably due to differences in sampling strategies and sample sizes. In contrast, the proportion of HIV-positive women observed aligns with national estimates for the general population in the DRC (1.2%, 95% CI: 0.08–0.16) [33]. The prevalence of HR-HPV infection that we found is similar to that reported by other Congolese researchers [25,27]. Indeed, while the global prevalence of cervical HPV infection is estimated at 9.9% [8], substantially higher rates have been documented in SSA [7,8], likely reflecting limited vaccination coverage and low screening rates [16,41].

It is well established that HbAS individuals possess a protective immune advantage against malaria [19,20], but our data suggest that this does not necessarily extend to a modified immune response to mucosal viral pathogens like HPV. This contrasts with other immunodeficiencies, such as HIV, systemic lupus erythematosus or solid organ transplantation, which significantly accelerate HPV-related carcinogenesis [18,42,43].

A meta-analysis of HPV isolate genomic sequences from cervical smears in African countries [24] showed that HR-HPV accounted for 66% of all HPV infections. However, data from the DRC were not included in the GenBank database used.

Although 72 smears that tested positive for HR-HPV were not sequenced, HPV16 and HPV18 were far from being among the four most common genotypes. The Cobas test detected HPV types 16 and 18 in 25 and 22 smears respectively, while HPV types 35, 52, and 31 were identified in 26–32 sequenced samples. An initial study found that HPV types 52, 58, 16, and 31 were the leading genotypes in samples from female sex workers in Kisangani [26]. In Kinshasa/RDC, the four most common HR-HPV types are 68, 58, 52, and 53 [25]. Other studies conducted in the country [27,44,45] did not perform HPV genotyping. Our findings align with the prevailing presence of HPV types 35 and 52 in SSA countries [13,46,47]. HPV35 is recognized as a major genotype that contributes to precancerous lesions and cervical carcinomas in Africa [46,48,49]. However, the Gardasil-9 vaccine does not protect against HPV35 and is estimated to prevent only 52% of HPV-related cervical cancer cases in Central, West, and Southern Africa. Therefore, it is essential to include HPV35 in future HPV vaccine formulation, particularly for use in Africa and the DRC [48,49]. The introduction of the quadrivalent HPV vaccine in the DRC, scheduled for 2026, will undoubtedly be a significant step forward [50]. However, its effectiveness is limited due to the predominance of non-16 and non-18 HPV genotypes in the country.

HPV types 16 and 18, which are detected in most cervical cancers, are less prevalent in women in SSA [10] and Asia [11,12]. Nevertheless, there is a growing decline in the prevalence of these two HPV types in Europe due to vaccination [51,52]. China has shown a predominance of HPV52 and HPV58 [3,53], while in Korea, HPV53 is the most common genotype in HSIL lesions, followed by HPV16, 58, 52, and 68 [12].

Epidemiological studies have identified 220 HPV reference types [14]. They are divided into HR-HPV, pHR-HPV, and LR-HPV based on their oncogenicity [1,3]. HPV types 103 and 223, whose new variants were detected in our study, are Gamma-HPV, belonging to the gamma 22 and gamma 6 families, respectively, and are known to have cutaneous tropism. They are present in 90% of cases of cutaneous squamous cell carcinoma in patients with epidermodysplasia verruciformis [54,55]. Later, HPV103 has been detected in cervical-vaginal smears from women with normal and abnormal cervical cytology [56], while HPV223 has been found in the oral mucosa and skin [57]. The detection of these two Gamma-HPVs in the cervical-vaginal smears from our cohort suggests that much remains to be discovered about these two viruses, particularly in relation to their tropisms.

The profile of HR-HPV-positive women (Table 4) may be linked to the selection criteria we applied. In addition, unmarried women (single, widowed, or divorced) may be indirectly exposed because they are more likely to have multiple sexual partners than married women. This could increase the risk of sexually transmitted infections, particularly HIV, which is a known risk factor for HPV infection [4,37]. Approximately 50% of women contract HPV infection around three years after their first sexual intercourse, and the infection rate is highest between the ages of 20 and 29 [58]. Studies in the DRC had already confirmed high rates of HR-HPV among people under 35 [25,45].

HPV33 belongs to group 1 oncogenic virus [37,59]. It appears to be more virulent (Table 7) than HPV35, which is prevalent in our cohort. Studies have confirmed that African HPV types 16, 18, 33, and 45 are more virulent than other HPV variants [8,60]. The major cytological abnormalities or cancer indicate the persistence of an HR-HPV infection and its integration into the host genome [14,15]. This integration is a multifactorial process influenced by various host-, virus-, and environment-related cofactors [17,37]

Beyond the well-established oncogenic potential of HPV types 16 and 18, studies conducted in SSA, particularly in Burkina Faso and Tanzania, have also reported significant associations between HPV types 35, 52, and 58 and HSIL [47,61]. In Italy, Bruno MT et al. found that 50% of the HPV strains implicated in women over 50 with CIN2 + were neither 16 nor 18 [62].

In about 10% of cases, HR-HPV infection may progress to precancerous or cancerous cervical lesions within 10–20 years [14,15]. This natural story may explain the association we observed between women aged 45–54 years and major cytological abnormalities. The role of intravaginal plants in the development of cervical lesions has been mentioned by some authors in the DRC [25,28]. Some medicinal plants have shown protective effects in preclinical studies due to their antioxidant, anti-inflammatory, and antiproliferative properties [63,64]. However, at high concentrations, the local application of certain plants—rich in essential oils— [65], could cause irritations or superficial lesions of the mucosa, altering the integrity of the cervical epithelium, vaginal microbiota, and local immunity. This results in an increased susceptibility to HPV infections or their persistence [66,67].

The regression of HPV lesions is linked to a local cell-mediated immune response against early proteins. Viral infection activates cytokine production by keratinocytes, intraepithelial CD8 + T lymphocytes, and CD4 + T helper1 [1,68]. Protection against severe malaria in HbAS individuals results from the early lysis of parasitized red blood cells. However, there is primarily a complex immune response combining innate immunity (natural killer cells, macrophages) and adaptive immunity (antibodies, T lymphocytes) [69]. The mobilization of cellular immunity, although local in the first case and systemic in the second, could motivate further exploration of viral clearance in women with HbAS, particularly through a longitudinal study.

This study has several strengths. First, it is among the first to examine the impact of HbAS status on HR-HPV infection and associated cytological abnormalities in cervical smears, while accounting for HIV serostatus. Second, it assesses the correlation between HR-HPV genotypes and major cervical abnormalities in a larger sample size than that of a previous study conducted in Kisangani, DRC. Furthermore, the identification of two novel HPV variants (103-like and 223-like) represents an important additional finding.

### Study limitations

Despite his strengths, our study has some limitations. Indeed, the primary limitation is the failure to reach the predetermined sample size for HbAS women. This may have introduced a selection bias, which could result in limited statistical power, potentially. This could explain the lack of statistically significant correlation observed between HbAS status and HR-HPV infection or cytological abnormalities. This limitation turns out to be a strength as recruiting a sufficient number of HbAS people was a real challenge. They are generally asymptomatic and, in our case, their sickle cell status was previously unknown. Second, DNA sequencing failed in 36% of samples tested positive for HR-HPV via the Cobas 6800 system. Studies suggest that insufficient tissue and insufficient DNA, correlated with DNA purity or degradation, account for around 90% of failures, regardless of test design [70]. Incorrectly quantified and/or contaminated DNA can significantly impact a sequencing run [71]. Thus, HPV DNA sequencing can be influenced by sample adequacy and affected by intrinsic variability associated with collection. The operator’s expertise and the patient’s hormonal status are also to be considered [72]. In our series, sequencing failure could be primarily attributed to pre-analytical conditions, particularly the fact that the residual fluids remaining after cytological analysis and Cobas HPV testing contained an insufficient genome; as well as the operator variability. It’s possible that the DNA in some samples degraded during shipping or due to the time lag between the HPV Cobas test and sequencing. However, despite its performance, sequencing on the Oxford Nanopore platform has a few limitations [72]. Third, the exclusion of invasive cancer cases may limit the clinical implications of our results.

### Generalizability and implications for future research

These results from a cross-sectional study conducted in the single urban center of Kisangani, cannot be generalized to the broader, more diverse population of the DRC. Longitudinal studies involving larger cohorts of women with HbAS are needed to better assess the impact of this allele on HR-HPV infection and cervical lesions, compared to women without SCD. Additionally, a study identifying carriers of HbAS among a sample of HIV-positive women would provide a better understanding of the respective contributions of HIV infection and the HbAS status to the progression of cervical HR-HPV infections.

## Conclusion

Our study shows that the prevalence of cervical HR-HPV infection is high in Kisangani, DRC. HPV35, HPV52, and HPV31 are the three most common genotypes, and sequencing identified two novel variants of HPV103 and HPV223. Our findings also highlight that advanced age, HPV33 infection and intravaginal herbal use were the main factors associated with major cervical abnormalities. Although HbAS status has not been associated with HR-HPV infection or cervical lesions, further studies with larger sample sizes are needed to determine this association in women with HbSS.

## Supporting information

S1 Appendix Data collection form. It details the variables related to the characteristics of the respondents, the risk factors sought, and the results of sickle cell screening and cervical smear tests.
(PDF)

S2 Appendix Inclusivity in global research. Questionnaire completed by the research team to assess the inclusivity of the study design, implementation, and reporting in alignment with global health equity principles.
(DOCX)

S3 Appendix Detailed distribution of high-risk HPV genotypes among HbAS and HbAA women. (DOCX)

S4 Appendix Cytology results by HPV genotype. (DOCX)
