# Supplementary material for: Human papillomavirus genotypes and factors associated with major cervical smear abnormalities in a sickle cell endemic area of Kisangani, Democratic Republic of the Congo
Source: PLoS One. 2026 Jun 10;21(6):e0350982. doi: 10.1371/journal.pone.0350982 (PMC13252769; doi:10.1371/journal.pone.0350982)
Supplement: S1 Appendix — It details the variables related to the characteristics of the respondents, the risk factors sought, and the results of sickle cell screening and cervical smear tests. (PDF) [file pone.0350982.s001.pdf]

FACULTY OF MEDICINE AND PHARMACY/ KISANGANI UNIVERSITY

DEPARTMENT OF GYNECOLOGY AND OBSTETRICS

DATA COLLECTION FORM

No. **Date:**

CERVICAL INTRAEPITHELIAL NEOPLASIA AND HUMAN PAPILLOMAVIRUS INFECTION IN KISANGANI, DRC: INFLUENCE OF SICKLE CELL ALLELE CARRIER STATUS

I. Identification

1. Name:
2. Age (Years):
3. Physical address:
   - Municipality……………………………… / Kisangani
   - ……………………………… (location) / Outside Kisangani
4. Phone:
5. Marital status
   - Single ☐
   - Monogamous marriage ☐
   - Polygamous marriage ☐
   - Divorced ☐
   - Widowed ☐
6. Level of education
   - None ☐
   - Pimary school ☐
   - Secondary ☐
   - High education and university ☐
7. Profession:
8. Religious affiliation:

II. Gynecological and obstetric history

9. Date of last menstrual period (LMP):

10. Age at first intercourse: ……… years

11. Parity (Number of deliveries): …………… ……………………………………………………………

12. P G A +

13. Age at first childbirth, if applicable (years):………………………………………………………….

14. Number of sexual partners during reproductive life:

- One ☐
- ≥ Two ☐

15. Number of sexual partners in the 12 past months:

- One ☐
- ≥ Two ☐

16. Episodes of cervicovaginitis (leukorrhea) treated in the last 12 months:

17. Previous cervical cancer screening

- No ☐
- Yes ☐
    - *Number of times:*
    - *When (last time):*
    - *Methods (biopsy, Pap smear, HPV test):*
    - *Hospital name / City name:*

18. History of sexually transmitted infections (gonorrhea, syphilis, etc.) in the 6 past months:

- Yes ☐
- No ☐

III. Medical risk factors:

- Chronic illness
    - Yes, What disease……………………………………………………………………………….
    - No ☐
- Use of intravaginal plants
    - Yes ☐ Name of the plant…………………………………………….
      Reason for use:………………………………………………………………………………
    - No ☐
- Ongoing medical treatment (*name of medication*)
  ……………………………………………………………………………………………………….
- History of oral contraception
    - None ☐
    - Occasional ☐
    - More than 3 months ☐

- Alcohol consumption
  - None ☐
  - Occasional ☐
  - More than 1 bottle per day ☐

IV. Interview

- Complaints/symptoms
  - None (participation in the study) ☐
  - Contact bleeding ☐
  - leucorrhea ☐
  - Other (to be completed)……………………………………
- Management
  - Cervicitis (treat before sampling) ☐
  - Suspected neoplasm: Biopsy ☐
  - Screen for precancerous lesions immediately ☐

V. Screening

- **Sickle cell screening result (HbAA, HbAS, HbSS)**

**Type of test**	**Result**
SC Hemotype (Rapid Test)	
liquid chromatography coupled with mass spectrometry(LC-MS)	

- **HIV screening (**according to the algorithm in effect in the DRC**):** *Yes / No*

**HIV serostatus**	
HIV-positive	
HIV-negative	

- **Cervical smear results** (after cytological analysis)

ASC-US	
ASC-H	
Low-grade SIL (LSIL)	
High-grade SIL (HSIL)	
Squamous cell carcinoma	
Adenocarcinoma in situ	

Other (specify)	

- **High risk HPV test result** after sample analysis

Negative HPV test	
Positive HPV test	
HPV type: if positive (sequencing)	

VI. Identification

- Hospital :……………………………………………………………………………………………………….
- Date of sample collection:……………………………………………………………………………….
- Collected by (names of team members)
  - Pap smear:…………………………………………………………………………………….
  - TDR/DREPA and HIV serostatus:……………………………………………………..

The Department of Obstetrics and Gynecology at the University Clinics thanks you for your participation in the study

- Principal Investigator: Dr. Neema Ufoy Mungu Yvette
- Supervisors: Prof. Dr. Katenga Bosunga and Prof. Dr. Juakali Sihalikyolo
