# Supplementary material for: Human papillomavirus genotypes and factors associated with major cervical smear abnormalities in a sickle cell endemic area of Kisangani, Democratic Republic of the Congo
Source: PLoS One. 2026 Jun 10;21(6):e0350982. doi: 10.1371/journal.pone.0350982 (PMC13252769; doi:10.1371/journal.pone.0350982)
Supplement: S2 Appendix — Questionnaire completed by the research team to assess the inclusivity of the study design, implementation, and reporting in alignment with global health equity principles. (DOCX) [file pone.0350982.s002.docx]

**Inclusivity in global research**

PLOS’ policy on inclusivity in global research aims to improve transparency in the reporting of research performed outside of researchers’ own country or community and ensures that PLOS publications reporting global research adhere to high standards for research ethics and authorship. Authors of relevant research articles may be asked to complete the questionnaire below, which outlines ethical, cultural, and scientific considerations specific to inclusivity in global research. This questionnaire may be requested when researchers have travelled to a different country to conduct research, if research uses samples collected in another country, research with Indigenous populations or their lands, or if research is on cultural artefacts. Researchers travelling to another country solely to use laboratory equipment will not normally be required to complete the questionnaire. However, the questionnaire can be requested at the journal’s discretion for any submission – if you have been requested to complete this questionnaire by the PLOS journal you submitted to, please do so.

Please complete the questionnaire below and include this as a Supporting Information file with your manuscript. Note that if your paper is accepted for publication, this checklist will be published with your article in the supporting information files. Please ensure that you reference the checklist in the main body of your manuscript. We suggest adding a subsection ‘Inclusivity in global research’ to your Methods section and adding the following sentence: “Additional information regarding the ethical, cultural, and scientific considerations specific to inclusivity in global research is included in the Supporting Information (**S4 Checklist**)”.

The questions have been designed to be applicable to a wide range of study types, and there are subsections for both human subjects research and non-human subjects research. If any of the questions are not relevant to your research please mark them as “N/A” as appropriate.

**Ethical considerations, permits and authorship**

*This section is applicable to all research types.*

Provide details as to who granted permissions and/or consent for the study to take place in the Methods section of your manuscript. This should include the names of **all** ethics boards, governmental organizations, community leaders or other bodies that provided approval for the study.

If individuals provided approval refer to these people by their role or title but do not list their name(s).

**Reported on page number 14:**

Locally, we obtained approval from the ethics committee of the University of Kisangani (approval no. UNIKIS/CER/023/2022) and, at the national level, from the School of Public Health of the University of Kinshasa (approval no. ESP/CE/65/2025) in the Democratic Republic of Congo (DRC). The Faculty of Medicine and Pharmacy at the University of Kisangani/DRC gave us research authorization, which we attached to the first approval from the ethics committee before visiting the various hospitals. The faculty research authorization was signed by the vice dean in charge of research.

Prior to data collection, and in addition to the authorizations already obtained, we sought and obtained the agreement of the chief medical officer of the provincial health division for the province of Tshopo, as well as the medical management of each hospital (i.e., the medical director and the administrative manager) that served as a study setting. They had all received a copy of the study protocol, which they had read and approved.

At each site, we sought and obtained agreement from the chefs of the streets where the various hospitals are located.

If there were any deviations from the study protocol after approval was obtained please provide details of these changes in the Methods section of your manuscript.

**Reported on page number 14:**

There were no changes after obtaining additional approval. Nevertheless, the initial protocol approved by the ethics committee of the University of Kisangani in December had been expanded. The initial research protocol had planned to limit itself solely to partial genotyping of high-risk human papillomaviruses (HR-HPV). The initial results grouped HR-HPV detected as HPV 16, HPV 18, and other types of HR-HPV. We decided to enhance our protocol by identifying other high-risk HPV types, types 16 and 18, and by seeking to identify the sites of integration of the viral genome into the host genome. This allowed us to better analyze the correlation between HR-HPV types and the cervical cytological abnormalities observed. For this reason, we submitted our protocol for additional approval by the ethics committee of the School of Public Health at the University of Kinshasa (capital of the DRC) . The change in the ethics committee was due to the fact that:

(i) The plenary sessions of the ethics committee of the School of Public Health in Kinshasa are more frequent (every month) than those at the local level (every six months);

(ii) The types of HPV diagnosed may have an impact on the course of action to be taken. Therefore, the results of our study could better contribute to the choice of vaccine type to be considered for primary prevention of cervical cancer in the DRC.

(iii) There was a change in all the members of the ethics committee of the University of Kisangani, and the handover was not yet effective when we made a change to our study protocol.

Did this study involve local collaborators that are residents of the country where the research was conducted or members of the community studied? If you do not have any authors from said communities, please provide an explanation for this below.

Everyone listed as an author should meet PLOS’ criteria for authorship and all individuals who meet these criteria should be included in the author byline, rather than the acknowledgements. For further information please see the journal’s Authorship Policy.

Yes, this study involved local collaborators residing in Kisangani ,in the DRC (where the study was conducted). This is confirmed by the authors' affiliation.

Three authors of this publication are members of staff at the University of Kisangani: The head of the department of gynecology and obstetrics at the University Clinics of Kisangani was involved in the design and supervision of the study; he also reviewed the manuscript and approved the final version. He is a co-author. The principal investigator of the study, who is also the first author, is a permanent resident of Kisangani, DRC. The second co-author, residing in Kisangani, was a doctor from another province of the DRC who had been specializing in Kisangani for three years. He participated in data collection and reviewed and validated the final version of the manuscript.

2. Almost all members of the research team were from the local community (doctors, nurses, and laboratory technicians). Only one of the two study supervisors was from the University of Liège in Belgium.

3. On the recommendation of street chiefs, we sought and obtained the participation of community relays in raising awareness to improve community adherence. These are people from the community, trained by health zones, who relay important information to the population in a health area, particularly during the distribution of mosquito nets, vaccination campaigns, or simply educational messages during epidemics.

**Human subjects research (e.g. health research, medical research, cross-cultural psychology)**

Did you obtain written informed consent from a representative of the local community or region before the research took place? How did you establish who speaks for the community? Details of written informed consent obtained from study participants should be reported separately in the Methods section of your manuscript.

Yes, we obtained written informed consent from the street chiefs in the different regions where the hospitals are located, as well as from the president of the Kisangani sickle cell association. We found the contact details of the street chiefs at the municipal administration. We contacted the Gracia Foundation (an association for sickle cell patients) to determine who could speak on behalf of people carrying the sickle cell allele.

Details regarding the written consent obtained from study participants are described in the methods section, specifically in the subsection entitled “participants” on page 7.

How did members of the local community provide input on the aims of the research investigation, its methodology, and its anticipated outcome(s)?

Community members participated in several ways and at different stages

o **During the design phase of our research** **project**: we consulted with the representative of the sickle cell association in Kisangani, as well as several women's associations, to gather their opinions on sickle cell disease and cervical cancer screening in the city of Kisangani during an informational seminar.

o **Awareness raising and invitation to screening**: We visited local churches and appeared on the local station of Congolese national radio and television to inform the population about the study and explain the benefits of screening. We also designed a number of posters about the study and displayed them in strategic locations around the city.

o **Methodology**: We used participatory methods. At the suggestion of street chiefs and hospital medical directors, we involved community representatives in raising awareness. They walked the streets with microphones three times a week to invite women to take advantage of free gynecological consultations with screening for cervical cancer and sickle cell disease.

o **Assessment of local needs**: Our research project was motivated by the lack of a screening program for cervical cancer and sickle cell disease in the DRC in general, and in Kisangani in particular. Street chiefs and women's associations expressed the wish that sickle cell disease be screened for in the children of participants who so desired, that gynecological consultations be supplemented by ultrasound examinations, and that sick people be treated. Taking into account these requests and the budget at our disposal, the children of AS women were screened on request, gynecological ultrasound screening was performed for all women for whom it was essential or on request, and vaginal ovules were administered free of charge to respondents with acute cervicitis. High-grade cervical lesions were treated free of charge, and the cost of surgical procedures for other diseases diagnosed during the campaign was reduced by 50%.

**o Communication with community representatives**. Throughout the data collection process, we held regular meetings with street chiefs to keep them informed of the study's progress, as well as with community relays who reported back to us in real time on the local population's reaction during awareness-raising activities in the streets.

When engaging with the local community, how did you ensure that the informed consent documents and other materials could be understood by local stakeholders?

To ensure that the informed consent forms and other documents (including participant information sheets) were clearly understood by all stakeholders, we translated them into the local languages: lingala and swahili. The original french document was available in the local language, and we spoke with each participant in the language of their choice (french, lingala, or swahili). We gave them the opportunity to express their concerns during our discussions and had prepared a few questions to test their understanding when no questions were asked.

The participants had the opportunity to ask clarifying questions during the general information sessions in the waiting room, before signing the consent form and before undergoing cervical smears and blood tests, and even at the time of communicating the results of the analyses of their samples. We also asked them a few questions to verify their knowledge before receiving the written consent forms, in order to correct any information that had been misunderstood.

Will the findings of the research be made available in an understandable format to stakeholders in the community where the study was conducted (e.g. via a presentation, summary report, copies of publications, etc.)? Please provide details of how this will be achieved.

**Yes, the research results will be made available to stakeholders in the community. We have considered four ways of doing this:**

**1. Collaboration with local organizations**:

If our article is accepted, we will share a copy of our publication with the provincial representatives of the national cancer control program, the sexually transmitted disease control program, and the national sickle cell disease control program. Through these government organizations, the results of the study can be used to initiate concrete actions in the fight against these diseases.

2. **Community meetings**: We will organize meetings with the same street chiefs, community representatives, and women's associations that contributed in one way or another to the design and implementation of the study to present the research results and gather their feedback.

3. **Brochures**: We will develop simple brochures with key findings and educational messages;these brochures will be given to community leaders and women's associations for dissemination.

4. **Dissemination through local media**: We will seize every opportunity that arises, including invitations received in connection with World Cervical Cancer Day, as an opportunity to educate the population and disseminate the relevant results of the study.

**Non-human subjects research using specimens/ animals collected as part of the study, or those housed in archival collections. Examples include archaeology, paleontology, botany and zoology.**

Did the permission you obtained from a local authority to perform the study include an agreement on access to outputs and benefit sharing? This may include procedures to enable fair distribution of the benefits and resources arising from the research performed. Please include any details of Prior Informed Consent and Benefit Sharing Agreements obtained. These may be required by field-specific regulations, for example the Convention on Biological Diversity (CBD) and the associated Nagoya Protocol.

**Not applicable**

If the material used in your study was imported, please A) provide the year it was imported and B) indicate whether permits were obtained to import/export the materials used, C) provide details of any permits obtained. If this information is not available, please indicate this.

**Not applicable**

If you used archival specimens, please state how the material used in your study was acquired by the institute it is held in and provide details of any permits obtained for the original excavations/ sample collection. If this information is not available, please indicate this.

**Not applicable**

How was the potential cultural significance of the materials collected in your study to local communities considered in your research design? Were Indigenous peoples and/or local researchers and institutions involved with archaeological excavations / collection of specimens? If so, please provide a description of their involvement.

**Not applicable**

If your manuscript includes photographs of human remains please indicate whether authors obtained permission from descendants or affiliated cultural communities to do so.

**Not applicable**
