# Supplementary material for: Human papillomavirus genotypes and factors associated with major cervical smear abnormalities in a sickle cell endemic area of Kisangani, Democratic Republic of the Congo
Source: PLoS One. 2026 Jun 10;21(6):e0350982. doi: 10.1371/journal.pone.0350982 (PMC13252769; doi:10.1371/journal.pone.0350982)
Supplement: S3 Appendix — (DOCX) [file pone.0350982.s003.docx]

| High-risk HPV genotypes | Total = 128  N (%) | HbAA (98) (n,%) | HbAS (30)  (n,%) |
| --- | --- | --- | --- |
| HPV16 | 14 (10.9%) | 9 (9.2%) | 5 (16.7%) |
| HPV18 | 16 (12.5%) | 13 (13.3%) | 3 (10.0%) |
| HPV31 | 26 (20.3%) | 20 (20.4%) | 6 (20.0%) |
| HPV33 | 19 (14.8%) | 11 (11.2%) | 8 (26.7%) |
| HPV35 | 32 (25.0%) | 20 (20.4%) | 12 (40.0%) |
| HPV39 | 14 (10.9%) | 12 (12.2%) | 2 (6.7%) |
| HPV45 | 12 (9.4%) | 9 (9.2%) | 3 (10.0%) |
| HPV51 | 10 (7.8%) | 6 (6.1%) | 4 (13.3%) |
| HPV52 | 27 (21.1%) | 21 (21.4%) | 6 (20.0%) |
| HPV56 | 18 (14.1%) | 17 (17.3%) | 1 (3.3%) |
| HPV58 | 21 (16.4%) | 15 (15.3%) | 6 (20.0%) |
| HPV59 | 9 (7.0%) | 9 (9.2%) | 0 (0.0%) |
| HPV66 | 13 (10.2%) | 9 (9.2%) | 4 (13.3%) |
| HPV68 | 23 (18.0%) | 18 (18.4%) | 5 (16.7%) |
| HR-HPV Gardasil 9 | 81 (63.3%) | 61 (62.2%) | 20 (66.7%) |

**Appendix S3. Detailed distribution of High-risk HPV types among HbAS and HbAA women**
