# Supplementary material for: Human papillomavirus genotypes and factors associated with major cervical smear abnormalities in a sickle cell endemic area of Kisangani, Democratic Republic of the Congo
Source: PLoS One. 2026 Jun 10;21(6):e0350982. doi: 10.1371/journal.pone.0350982 (PMC13252769; doi:10.1371/journal.pone.0350982)
Supplement: S4 Appendix — (DOCX) [file pone.0350982.s004.docx]

| HR-HPV Types (n) | NILM  (n,%) | Inflammatory smears (n,%) | ASC-US (n,%) | LSIL  (n,%) | AGC-NOS,  AGC-ecc Neo  (n,%) | ASC-H, HSIL  (n,%) | Invalid smears  (n,%) |
| --- | --- | --- | --- | --- | --- | --- | --- |
| HPV16 (14) | 3 (21.4%) | 3 (21.4%) | 4 (28.7%) | 2 (14.3%) | 0 (0.00%) | 1 (7.1%) | 1 (7.1%) |
| HPV18 (16) | 7 (43.8%) | 5 (31.3%) | 1 (6.2%) | 1 (6.2%) | 0 (0.0%) | 2 (12.5%) | 0 (0.0%) |
| HPV31 (26) | 13 (50.0%) | 7 (26.9%) | 4 (15.4%) | 1 (3.8%) | 0 (0.0%) | 1 (3.8%) | 0 (0.0%) |
| HPV33 (19) | 11 (57.9%) | 1 (5.3%) | 2 (10.5%) | 2 (10.5%) | 2 (10.5%) | 1 (5.3%) | 0 (0.0%) |
| HPV35 (32) | 14 (43.7%) | 5 (15.6%) | 7 (21.9%) | 3 (9.4%) | 0 (0.0%) | 3 (9.4%) | 0 (0.0%) |
| HPV39 (14) | 9 (64.3%) | 0 (0.0%) | 4 (28.6%) | 1 (7.1%) | 0 (0.0%) | 0 (0.0%) | 0 (0.0%) |
| HPV45 (12) | 8 (66.7%) | 3 (25.0%) | 1 (8.3%) | 0 (0.0%) | 0 (0.0%) | 0 (0.0%) | 0 (0.0%) |
| HPV51 (10) | 3 (30.0%) | 2 (20.0%) | 4 (40.0%) | 0 (0.0%) | 0 (0.0%) | 1 (10.0%) | 0 (%) |
| HPV52 (27) | 13 (48.2%) | 7 (25.9%) | 4 (14.8%) | 1 (3.7%) | 0 (0.0%) | 2 (7.4%) | 0 (0.0%) |
| HPV56 (18) | 8 (44.4%) | 5 (27.8%) | 2 (11.1%) | 2 (11.1%) | 0 (0.0%) | 0 (0.0%) | 1 (5.6%) |
| HPV58 (21) | 11 (52.4%) | 3 (14.3%) | 1 (4.8%) | 4 (19.0%) | 0 (0.0%) | 2 (9.5%) | 0 (0.0%) |
| HPV59 (9) | 4 (44.4%) | 0 (0.0%) | 4 (44.4%) | 0 (0.0%) | 0 (0.0%) | 0 (0.0%) | 1 (11.1%) |
| HPV66 (13) | 5 (38.4%) | 1 (7.7%) | 5 (38.4%) | 1 (7.7%) | 0 (0.0%) | 1 (7.7%) | 0 (0.0%) |
| HPV68 (23) | 15 (65.2%) | 3 (13.0%) | 5 (21.8%) | 0 (0.0%) | 0 (0.0%) | 10 (0.0%) | 0 (0.0%) |

**S4 Appendix. Cytology results by HPV genotype**

One sample that tested positive for HPV33 revealed the presence of both glandular (AGC-ecc, favor neoplastic) and squamous lesions (HSIL).
